# CRISPRing through time: How cutting-edge technology is revolutionizing life sciences and medicine

**DOI:** 10.1016/j.omtn.2026.103003

**Published:** 2026-07-07

**Authors:** Ishani Banik, Jean-Philippe Coppé

**Affiliations:** 1 Department of Radiation Oncology, University of California, San Francisco, San Francisco, CA, USA

**Keywords:** MT: RNA/DNA Editing, CRISPR-Cas systems, genome editing, functional genomics, precision medicine, computational approaches, modern-day sequencing efforts, public trust, computational biology, programmable nucleic acid platform

## Abstract

Given the plethora of emerging technologies, none have truly captured the minds as CRISPR. From the groundbreaking research, the ultimate battle of the prizes and patents to a number of books, the science of CRISPR continues to be significant in the biomedical field. For many decades now, the emergence of synthetic biology as an intervention to correct diseases has become the foundation of biomedical research. Clustered Regularly Interspaced Short Palindromic Repeats (CRISPR)-based genetic editing has become a common place for routine investigation of scientific hypotheses in pre-clinical settings. More recently, CRISPR-based diagnostic testing kits for SARS-CoV-2 have showcased a translational output. Furthermore, a technological landmark was achieved when the Food and Drug Administration (FDA) approved the first CRISPR-based gene therapy (exa-cel) to edit erythroid specific enhancer region of *BCL11A* in hematopoietic stem cells, introduced in patients suffering from sickle cell anemia to achieve durable remission. In this review, we provide a snapshot into the most important milestones along the journey of CRISPR from its discovery in bacteria to its usage in precision medicine. The intervention of machine learning tools has now intertwined complex biology with high-throughput scalable outputs. Given the vast amount of information on CRISPR, we try to pin down key take-home messages for scientists as well as non-scientist readers. This review article attempts to understand why and how CRISPR remains significant and seamlessly integrates in the emerging era of new technologies.

## Graphical abstract

**Figure:**
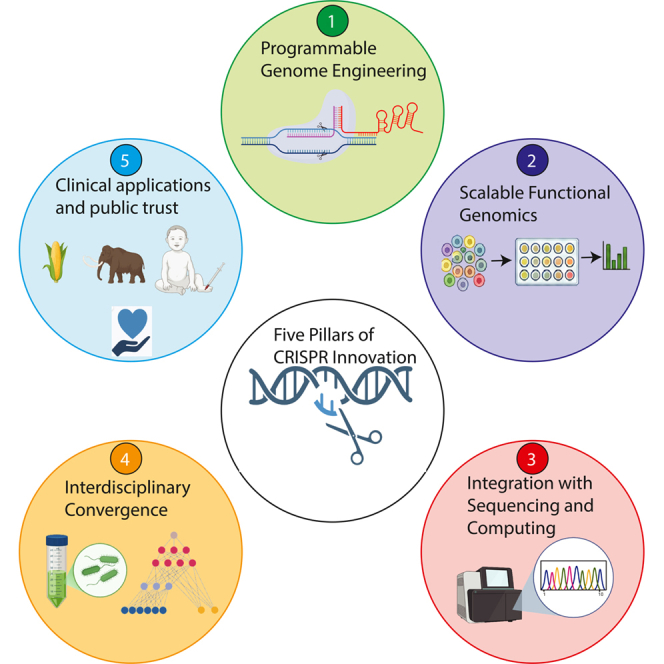

## Teaser

Banik and Coppé provide a snapshot into the most important milestones along the journey of CRISPR from its discovery in bacteria to its usage in precision medicine and its integration with computational biology modern day tools. The authors try to pin down key take-home messages for scientists and non-scientist readers.

## Introduction

CRISPR-based technologies have rapidly transformed biological research, enabling precise and scalable interrogation of gene function across diverse systems. At its core, CRISPR technology functions as a programmable nucleic-acid-guided platform, in which RNA-directed targeting is coupled with sequencing-based readouts to enable precise and scalable interrogation of gene function. This review begins with the biological foundations of CRISPR-mediated immunity and traces the key discoveries that established CRISPR-Cas9 as a programmable genome editing tool. We then examine how these advances catalyzed a new era of functional genomics, including high-throughput genetic screens and disease modeling approaches that surpass earlier perturbation technologies. The review further explores the integration of CRISPR with next-generation sequencing and computational methods, particularly in the context of single-cell genomics. Finally, we discuss the translation of CRISPR technologies into clinical applications, alongside emerging ethical considerations and the importance of public trust in shaping their future use. In this review, we outline five key reasons why CRISPR technologies have transcended the boundaries of traditional science, spanning their programmability, scalability, multi-modal versatility, integration with computational and sequencing frameworks, and rapid clinical and societal translation.

### The birth of genome engineering

The biological information of an organism is stored in its DNA. Every cell in the body carries a bundle of DNA that codes for about 30,000 genes. The genetic blueprint of an individual reflects their hereditary nature of phenotypic traits including diseases. After the advent of DNA sequencing, the causative gene(s) of debilitating disorders became widely accessible. Over 100,000 mutations have been identified in association to diseases so far and many more remain to be identified with ongoing research.^1^ The recognition that correcting for these specific genetic alterations that cause subtle changes in the disease-causing gene emerged as a field of synthetic biology. This is a concept where molecular biology tools are used for genetic editing in order to abrogate particular genomic abnormalities in particular cancers. Today, the availability of precise genome editing sets the stage for cure like never seen before. One of the genetic editing tools approved for human intervention is CRISPR-based therapies.

A number of pre-clinical attempts must be made before genome editing can be tried on humans. Prior to the advent of CRISPR, genetic editing tools included TALENs (transcription activator-like effector nucleases) and ZFNs (zinc finger nucleases).^2^ A complete review on the evolution of genome engineering can be found in another study.^3^ Soon after the discovery of the DNA double helix^4^ and restriction endonucleases,^5^^,^^6^^,^^7^ genetic engineering was born. With it arouse the prospect of gene therapy—a therapeutic strategy to use genetic engineering to treat diseases.^8^^,^^9^ The early 1960s saw the first attempts of gene therapy using recombinant DNA, which was further developed using other genetic engineering tools like viral vectors.^10^^,^^11^ Another early method of gene editing was the Cre/*loxP* system^12^^,^^13^ that was used for genetic modifications in mice albeit with low efficiency of homologous recombination. DNA is prone to damage such as breaks in their double strand, leading to mutations. Homologous recombination is a double-stranded break repair mechanism that involves the exchange of DNA between two identical or highly similar DNA regions. An advancement in the technology was made with the discovery of ZFNs that could overcome the low homologous recombination efficiency by introducing a double-strand break at a target site.^14^^,^^15^ A further improvement was made with TALENS that allowed for introduction of double-stranded breaks at an even higher efficiency and a simpler design.^16^^,^^17^^,^^18^ However, the problem that persisted was the need to engineer a new protein for each target until the discovery of CRISPR-Cas9 gene editing systems. The CRISPR-Cas9 system did not rely on complex engineered proteins for DNA binding. Instead, it used an easily programmable single guide RNA (sgRNA) to bind to the Cas9 enzyme that would guide the Cas9 to the desired site of double-stranded break in the DNA.

The palindromic repeat sequences of CRISPR were first identified in the DNA of *Escherichia coli* bacteria.^19^ Several years later, another study observed similar patterns of repeated sequences in *Haloferax mediterranei*.^20^ The similarity of sequences across prokaryotes like bacteria and archaea hinted toward a conserved evolutionary mechanism and the possibility of its role in DNA repair or gene regulation. Several key observations in the origin of spacer sequences found in CRISPR and the presence of nuclease and helicase domains in Cas (CRISPR-associated genes) led to the prediction that CRISPR-Cas is an adaptive defense system in bacteria that uses antisense RNA memory signatures.^21^ The prediction was deemed accurate when a team from a yogurt making firm-Danisco reported that the removal or addition of specific spacers altered resistance to their starter culture of bacteria when challenged with a virus.^22^ Most importantly, it was shown for the first time that the target of immune system of prokaryotes was foreign DNA and not mRNA,^23^ resulting in priming of the scientific community that could now harness this naturally occurring phenomenon as a powerful tool in lab. This RNA-guided recognition mechanism forms the basis of CRISPR as a programmable nucleic-acid-targeting system, enabling precise identification and modification of complementary DNA or RNA sequences. One of the first applications for CRISPR was patented by Danisco on bacterial vaccination by observing that their starter culture of bacteria developed acquired resistance against bacteriophage predation when new CRISPR-derived spacers were introduced in their bacterial cultures. The immunization process is based on incorporating short DNA sequences from virulent phages into CRISPR locus, the transcripts of which give rise to a protein complex that recognizes and cleaves foreign DNA.^24^

### The biology of CRISPR

Proteins associated with the prokaryotic CRISPR system can be assigned to either the adaptive module or the effector module. The CRISPR-Cas system is presently classified into two classes and six types.^25^ The classical CRISPR system employs Cas9, categorized as class 2; and type II, Cas12, categorized as type V; and Cas13, categorized as type VI. All of the three Cas modules are defined by the use of a single multidomain effector protein, making them particularly amenable to genome engineering applications.

Unlike tandem repeats in the genome, CRISPR repeat clusters are separated by non-repeating DNA sequence called spacers. The CRISPR repeat clusters or sequences are found in 40%–50% bacteria and 85%–90% archaea.^26^ These CRISPR repeat clusters and spacers are adjacent to a well-conserved family of genes called CRISPR-associated genes, i.e., Cas. A critical discovery made in yogurt cultures that develop resistance to bacteriophages was the fact that spacer sequences of CRISPR dictate the specificity of Cas enzymes, which provide resistance against bacteriophages.^22^ It was further observed that the activity of Cas enzymes is guided by short CRISPR RNA (crRNA) transcribed from the spacer sequences.^27^ The transactivating crRNA (tracrRNA), a small RNA upstream of type II CRISPR-Cas locus in *Streptococcus pyogenes*, was reported to be essential for crRNA maturation.^28^ One key finding was the similarity of the acquired spacer sequences; these regions were called protospacer-adjacent motifs (PAMs). Whether the entry of an element is foreign or not is decided by the presence of PAMs. Those DNA sequences stored in the CRISPR locus as spacers and not containing PAMs are not recognized as foreign and not attacked by the prokaryotic immune system.^29^ Hence, PAMs, short sequence motifs adjacent to the crRNA-targeted sequence in invading DNA, are crucial for adaptation and interference in type I and type II CRISPR systems.^3^ Although there are six types of CRISPR system based on Cas modules, type II Cas9 turned out to be the most relevant due to its unique property of utilizing a single protein for RNA-guided DNA recognition and cleavage.^30^^,^^31^ Beyond Cas9, additional class 2 CRISPR systems have expanded the functional diversity of RNA-guided targeting.

To summarize, two landmark studies showed that ribonucleoprotein complexes preloaded with small, interfering Cas9-crRNA could act as a guide and target the degradation of a foreign nucleic acid. They demonstrated that Cas9 generates site-specific double-stranded breaks on DNA strands complementary to crRNA *in vitro*. It is the two-RNA structure formed from the mature crRNA base paired to tracrRNA that directs CRISPR-associated Cas9 to introduce double-stranded breaks in the target DNA. It was reported that the dual-tracrRNA:crRNA can be engineered as a sgRNA that is also capable of sequence-specific double-stranded breaks.^30^ Overall, there were two important aspects to foreign DNA recognition—base paring with crRNA and presence of PAM adjacent to target sequence of DNA.^3^ These studies highlighted the potential of this easily programmable RNA-guided DNA endonucleases. This innovation established CRISPR-Cas9 as a versatile nucleic-acid-guided editing platform, in which target specificity is encoded within a short RNA sequence rather than protein structure. Researchers realized that they could target any DNA sequence with an appropriate PAM sequence to introduce desired nicks through Cas9-mediated cleavage with a 20-nucleotide sgRNA. The specificity of the RNA-guided endonucleases could now be customized by replacing a short synthetic RNA molecule.^32^ This was an improvement in comparison to the tedious process of protein engineering previously used in ZFNs and TALENs. By piecing together the knowledge on CRISPR locus, Cas9 enzyme, its functionality using the dual tracrRNA:crRNA, and its reprogrammable facets using a single sgRNA, the stage was set for leveraging the CRISPR-Cas9 system for genome editing in eukaryotes.

Recently, Cas modules have been used in rapid detection of nucleic acids. These approaches utilize the non-specific endonuclease activity of Cas12 and Cas13 proteins to bind to a specific target via programmable crRNA. By combining the programmable specificity of Cas13 or Cas12 with a reporter molecule that is activated upon target recognition, these enzymes result in specific and sensitive indication of the presence or quantity of a nucleic acid.^33^ Cas12 endonucleases only require crRNA to create an efficient cut at single-stranded DNA (ssDNA) and double-stranded DNA (dsDNA). Notably, Cas12 exhibits collateral cleavage activity upon target recognition, enabling its use in nucleic acid detection platforms such as DETECTR.^34^ In contrast, Cas13 modules target RNA rather than DNA, enabling programmable RNA interference without permanent genomic modification. Cas13a and Cas13b modules are derived from *Leptotrichia shahii* bacterium (*Lsh*Cas13a) and *Prevotella* sp. (*Psp*Cas13b), respectively. Similar to Cas12, Cas13a requires single crRNA for its activation. Cas13b is more precise than Cas13a and is associated with mature crRNA and results in non-specific RNA cleavage.^35^ An application of Cas13a was its use in SHERLOCK (specific high-sensitivity enzymatic reporter unlocking) detection assays, widely used for detection of viruses, mutations, and pathogen screening.^33^ While Cas9 remains the predominant tool for genome editing, Cas12 and Cas13 provide complementary capabilities, including alternative cleavage patterns, expanded targeting scope, and applications in diagnostics and transient gene regulation. These platforms highlight the broader paradigm of CRISPR as a nucleic acid detection system, in which target recognition is coupled to signal amplification through enzymatic activity, enabling highly sensitive and specific diagnostic applications.

### The seminal discoveries of 2013 that shaped the future of CRISPR research

The work performed so far was on prokaryotic bacteria *S. pyogenes* and *Streptococcus thermophilus*. Scientists now used humanized versions of the Cas9 found in prokaryotes along with custom designed sgRNA. The lab of Dr. Feng Zhang demonstrated that precise cleavage at genomic loci in human and mouse cells can be achieved using Cas9 nucleases directed by short RNAs.^36^ Their group also showcased the possibility of multiplexing the technique by using several sgRNAs encoded into a single CRISPR array for simultaneously editing multiple sites in the genome.^36^ The simplicity of the technique, the lentiviral delivery of genome scale CRISPR-Cas9 knockout into the cells, and its representation were truly remarkable, given the fact that to this day most researchers use the freely available two-page protocol on the Lentiviral CRISPR tool box published by Dr. Feng Zhang.^37^ The group of Dr. George Church demonstrated that they were able to reach high CRISPR target rates in 293T cells, K562 cells, and induced pluripotent stem cells. They produced a genome-wide resource of ∼190,000 unique gRNAs targeting ∼40.5% of human exons, covering roughly 18,000 genes.^38^ The assembly of Cas9 with sgRNA resulting in the Cas9-RNA complex that induces the formation of double-stranded breaks in genomic DNA was the limiting factor for efficient DNA cleavage. The lab of Dr. Emmanuelle Charpentier worked on characterizing a large panel of tracrRNA and Cas9 orthologs, contributing to a database to improve the design of RNA-programable Cas9.^39^ Soon enough, the team of Dr. Jennifer Doudna reported on enhancing the DNA targeting activity *in vivo* by extension of the RNA sequence at the 3′ end.^40^ Other studies recapitulated the phenomena of high-frequency, site-specific genome modifications using sgRNA:Cas9 in human cells.^32^ The team of Dr. Keith Joung furthered these studies *in vivo*, by genomic modifications in zebrafish using sgRNA:Cas9. They reportedly succeeded in editing genomic sites that had previously failed with TALENs.^41^ Other studies corroborated the use of CRISPR-Cas9 system for the generation of gene-modified mice and zebrafish.^42^

CRISPR-Cas9-mediated genetic perturbations were simple, scalable, robust, and easily programmable, allowing researchers to study the linkage between genetic variations and disease phenotype in any organism of choice. This, in our opinion, accounts for the first reason on the profound impact the technology has had on science. What followed next was momentous speed in the field of biomedical research. This technology empowered scientists to mimic genomic translocations *in vitro* and *in vivo* as is often seen in tumor initiation and progression. It is worth mentioning that open-source distribution of plasmids carrying Cas9 by Addgene played an important role in the momentous speed of discoveries during this era.

### The utility of CRISPR system in the advancement of medical science

#### The advent of rapid disease modeling

One of the first experiments on successful genomic translocations involved the introduction of oncogenic driver events of lung cancer into HEK293T cells^43^ and t(11;22) translocation in human mesenchymal and stem cells.^44^ It was now possible to accurately model genetic events in cancer to improve our understanding of the underlying biology. The use of CRISPR-Cas9 accelerated the generation of animal models for the study of cancer. Even though the availability of small interfering RNA (siRNA)/short hairpin RNA (shRNA)-mediated genome perturbations and Cre/*loxP* systems were used by scientists, CRISPR rapidly superseded these techniques. Soon after the discoveries in 2013, genetically engineered mouse models of *ALK-1*-positive non-small cell lung cancer were generated using CRISPR-Cas9.^45^ Another study demonstrated that the technique could be used for efficient one-step insertion of fluorescent reporters in mice, thus introducing the idea of easily programmable and scalable generation of animal models that can carry reporters in endogenous genes.^46^ Some other notable examples include the delivery of Cas9-guided complexes targeting *Bth* allele that ameliorated hearing loss in the *Tmc1* mouse model,^47^ as well as the modeling of hepatocellular cancer, colon cancer, acute myeloid leukemia (AML), muscular dystrophy, and many other conditions. An extensive study on CRISPR-generated *in vivo* models has been reviewed elsewhere.^48^ *In vivo* models of cardiovascular disease, liver function, and Rett syndrome have been instrumental in advancing our understanding of disease mechanisms and evaluating potential gene editing therapies. Besides these models, the CRISPR system was used for functional screening studies to explore drug resistance in cancer.

#### Widespread availability of CRISPR functional screens

Lentiviral delivery of a genome-scale library—GeCKO library targeting 18,080 genes with more than a thousand unique guides—enabled positive and negative selection in human cells, leading to the identification of genes essential for cell viability in cancer and pluripotent stem cells.^37^ Additionally, the study also reported genes whose loss was associated with drug resistance in melanoma. Researchers started using genome-scale lentiviral sgRNA library to knock out pools of cells and then challenge cells with a specific drug to screen for survivors; such type of screen is called a positive selection screen. For example, thousands of unique guides (sgRNAs) were lentivirally delivered in *BRAF* mutant melanoma cells that were challenged with the *BRAF* inhibitor—vemurafenib. The cancer cells that survived the challenge were resistant to vemurafenib due to the loss of genes, including mainly *NF1* (i.e., loss of tumor suppressor function). A negative selection screen is when a pooled library of sgRNA is used to target genes essential for cell survival (*BCAR3* and *EGFR*, i.e., gain of oncogenic function).^49^ At the end of screen, those sgRNAs missing from the pool of cells are deemed as essential to cell survival.^37^ Other reports corroborated the use of pooled positive and negative sgRNA screens to evaluate genes essential for cell survival^50^ and those responsible for drug resistance.^51^^,^^52^

Pooled libraries such as the Brunello library for gene knockouts (CRISPRko), Dolecetto library for CRISPR interference (CRISPRi), and Calabrese library for CRISPR activation (CRISPRa) made genome scale interrogation in diverse model systems possible and publicly available to scientists. These libraries were an improvement when compared to the GeCKO libraries in terms of fewer sgRNAs required for the same perturbation level and had fewer off-target effects. Hence, a suite of genome-wide tools were established to efficiently interrogate gene function with multiple modalities.^53^

#### Beyond functional CRISPR screens

Next came the era of combinatorial screening; dual gRNA libraries were used to achieve dual gene perturbations. Within the dual gRNA libraries, each gRNA was designed to target either a gene or a scrambled non-targeting sequence absent from the genome. Thus, all combinations of gene-gene (double-gene perturbation) and gene-scramble (single-gene perturbation) were assayed for effects on cell growth.^54^ This combinatorial CRISPR screen was notably relevant for the study of synthetic lethality in *BRCA* mutations. Synthetic lethality describes the situation where a defect in one gene is compatible with cell viability but results in cell death when combined with another gene defect. *PARP1* inhibitors were the first to exploit the concept of synthetic lethality in regard to cancer cell death in *BRCA*-deficient tumors. *BRCA1*/*2* proteins execute the HR (homologous recombination)-mediated repair of damaged DNA and protect stalled replication fork. In cancer cells that have *BRCA* mutations, DNA damage repair can be alternatively performed through *PARP1*. Therefore, blocking both pathways of DNA repair—the presence of mutations in *BRCA* and the blockade of *PARP1*—implicates cell death in tumor cells. Therefore, *BRCA1*/*2*-deficient tumors were deemed sensitive to *PARP*1 inhibitors through the mechanism of synthetic lethality.^55^ CRISPR screens set the stage for identifying DNA-damage-related interactions such as those in *BRCA1*/*2*. Researchers could now identify genes that could rescue sensitivity to *PARP1* inhibition. They could even identify co-dependencies with *BRCA* (such as *RNASEH2A* and *RNASEH2B*) that may cause synthetic lethality.^56^^,^^57^^,^^58^ The scalability of CRISPR-based functional screens is fundamentally enabled by their nucleic-acid-based architecture, in which gRNA libraries serve as both perturbation agents and molecular barcodes. Coupled with next-generation sequencing, this allows direct quantification of guide RNA abundance and enables high-throughput, unbiased identification of gene function across the genome (Table 1).

**Table 1:** CRISPR technologies as nucleic-acid-guided platforms across functional and translational applications

Application area	CRISPR module	Nucleic acid component	Readout	Examples	Key advancement
Functional genomics	bulk (pooled) CRISPR screens	pooled sgRNA library (DNA constructs encoding guide RNAs)	NGS (guide abundance)	gene essentiality screens, drug resistance screens, saturation mutagenesis screens	NGS-enabled functional screening coupled with development of computational decomposition approaches
Functional genomics	single-cell CRISPR screens	barcoded sgRNA library compatible with scRNA-seq	scRNA-seq (transcriptome + guide identity)	Perturb-seq, CROP-seq	resolution of individual perturbation effects, distinguishing specific responses from bulk population-level signals
Disease modeling	CRISPR-Cas9 knockout/knockin	sgRNA ± repair templates (DNA donors)	targeted sequencing + phenotypic assays	*in vitro* and *in vivo* disease models	one-step insertion of reporter genes and programmable genome modification, superseding siRNA-/shRNA-based gene perturbation approaches
Diagnostics	CRISPR-Cas12/Cas13 systems	crRNA + reporter molecules	fluorescent- or signal-based detection	DETECTR, SHERLOCK	programmable nucleic acid detection with signal amplification
Therapeutics	base editing and related CRISPR-derived precision editors	sgRNA, mRNA, or DNA/RNA delivery constructs	genomic validation + clinical outcomes	*ex vivo* edited cells, *in vivo* genome editing	precise single-nucleotide modification without double-strand breaks, enabling targeted therapeutic intervention

#### Beyond base editing

A more versatile and precise method of genome editing that does not depend on double-stranded breaks was invented with prime editing. The prime editor (PE) complex consists of a prime-editing guide RNA (pegRNA) fused with an MMLV reverse transcriptase and Cas9 nickase (H840A), which is utilized to introduce specific edits at target loci.^59^ The Cas9 nickase used in PE is different from Cas9 nucleases that yield double-stranded breaks. Cas9 nickase (nCas9) is created by replacing one of the two nuclease domains in *Streptococcus* *pyogenesis* Cas9 (spCas9) producing nicks or single-stranded breaks. The nCas9 variant H840A cleaves non-target DNA strands.^60^ While traditional base editing were limited to C-to-T and A-to-G conversions, prime editing was developed to overcome these limitations by enabling all types of DNA substitutions, deletions, and insertions. Since base editing required making a double-stranded break, they were prone to high risk of unwanted targets and off-target effects. Since its invention, there have been several versions of PE, each with an improved efficiency for gene editing. These range from engineered pegRNAs (epegRNAs), introduction of mutations in nCas9 (H840A + N863A), reduction in the size of the PE complex (sPE), and engineered spCas9 variants to broaden the range of targetable sites limited by PAM sequence.^61^ Prime editing has been used in induced pluripotent stem cell (iPSC) model to correct single-exon mutation deletion in Duchenne muscular dystrophy (DMD).^62^ Other examples of therapeutic applications of prime editing include correcting single-base G-to-A^63^^,^^64^ and T-to-C^65^ transitions in mouse models of liver diseases. Current delivery methods include use of adeno-associated virus (AAV) systems and dual AAV systems where the vectors are introduced separately into the cell and reassembled.^61^ The primary challenge remains to be the achieving high efficiency and specificity across cell types along with the delivery of prime editing components *in vivo*.^66^

The prospect of editing and targeting the human genome-precision medicine was within reach and closer than ever before. Today, genome-wide CRISPR screens are being used to comprehensively map cancer dependencies in hundreds of cell lines like DRIVE^67^ and DepMap.^68^ The applicability of the CRISPR technology to study the function of a gene by loss of function (CRISPRko), defective function (CRISPRi), or gain of function (CRISPRa) in genetic screens alongside combinatorial screens while providing multiplexed interface for users to leverage has proven to be an efficient genetic editing tool kit. Moreover, the availability of multiplexed libraries for genome-wide perturbations and plasmids for single-gene modifications on Addgene as well as websites like chopchop.cbu.uib.no for gRNA selection has provided the ability to researchers across diverse expertise to try their hand at CRISPR. This, in our opinion, is the second reason why the technology has crossed beyond boundaries of traditional science.

### The influence of next-generation sequencing in the advancement of CRISPR technology

#### Early-stage sequencing efforts

The integration of next-generation sequencing (NGS) with CRISPR technology has enabled high-throughput and scalable readouts for genome editing experiments. In pooled CRISPR screens, NGS is required to quantify the abundance of sgRNAs depleted or enriched at the end of the selection. Short-read Illumina platforms like MiSeq and NextSeq were pivotal during the first studies of saturation mutagenesis screens because of their accuracy and scalability. Saturation editing is a powerful tool that enables functional analysis of nearly all possible single-nucleotide variants (SNVs) in a target gene. Findlay and colleagues were the first to report on saturation editing on all 13 exons of *BRCA1*.^56^ They used array-synthesized oligonucleotide pools containing all possible SNVs spanning each exon- SNV library. Target cells were co-transfected with the SNV library alongside Cas9/gRNA plasmid, and variant frequencies were quantified by sequencing of edited exon from guide DNA (gDNA) after selection. Illumina sequencing to quantify the frequency of each variant was followed by inference of variant functionality through customized bioinformatic pipelines. Although powerful, this approach was cumbersome, expensive, and technically demanding, requiring extensive computational infrastructure to analyze the sequencing data and assign functional scores to each variant. As a result, the field sought more accessible and rapid methods for evaluating CRISPR editing outcomes.

#### Entry of computational decomposition tools

With CRISPR-Cas9, several sgRNAs can be used at once in the case of pooled experiments, while many sgRNAs are needed to test the efficiency of the predicted targeted DNA sequence.^69^ Enzymatic Surveyor and T7 endonuclease I cleavage assays that were used to detect small changes in the DNA sequence of the target cells. However, these assays suffered from the detection of sequence polymorphisms,^70^^,^^71^ which resulted in difficulties to distinguish true edits. Another approach involved PCR amplification of the target region, followed by cloning and Sanger sequencing, which was accurate but labor-intensive and low throughput. Alternatively, in high-throughput screen such as the saturation mutagenesis screens of *BRCA1*, NGS analysis of target regions could be performed.

The advent of computational decomposition tools dramatically simplified this process. TIDE (tracking of indels by decomposition) was the first algorithm that facilitated the identification of major induced mutations in the targeted DNA site and its frequency in a cell population. This algorithm was provided as a web tool and a freely available R code, a much needed acceleration in terms of Sanger-sequencing-based tracing systems for pooled CRISPR studies.^72^ This represented a key shift toward democratizing genome editing analysis, offering a rapid, reproducible, and cost-effective alternative.

By now, these platforms provide automated analysis of indel composition, size distribution, and editing frequency, often requiring only basic molecular biology workflows and no high-throughput sequencing infrastructure. A recent comparison of these tools concluded that while TIDE and ICE^73^ (interference for CRISPR edits) are well suited for *in vitro* germline editing, tools like DECODR^74^ (deconvolution of complex DNA repair) may be better suited for *in vivo* or somatic CRISPR perturbations, particularly where greater sensitivity or heterogeneity is involved. Another powerful tool for amplicon-based NGS analysis is CRISPResso2,^75^ which was developed to provide more detailed assessment of editing outcomes, especially in pooled or multiplexed designs. However, it is still more computationally intensive than simpler Sanger-based tools and was not yet available during the earliest saturation editing studies.

In conclusion, the commencement of high-throughput CRISPR screens has showcased how Sanger sequencing and NGS can be used to convert a biological phenotype into a digital readout and map it back to its biological consequence. At the same time, tools like DECODR and TIDE have significantly lowered the barrier to entry, allowing broader use of CRISPR technology in labs without access to high-end sequencing platforms. The amalgamation of basic biology, NGS, bioinformatics, and a deep understanding of disease phenotype is bridging the gap between cutting edge research and everyday genome editing applications. This, in our opinion, is the third reason why CRISPR technology has changed how science can be accelerated.

### Combining single-cell sequencing with pooled CRISPR screens

#### Limitations of bulk sequencing

While NGS enabled the first wave of pooled CRISPR screens, it was limited in key areas. It could not resolve cell-to-cell heterogeneity, link sgRNA perturbation to multi-omic changes, or generate more than one phenotypic readout per experiment (such as survival). Despite these limitations, pooled CRISPR screening techniques such as dropout screens were pivotal in the discovery of essential genes,^37^^,^^50^ including genes involved in drug resistance^76^ and immune regulation.^77^ While these studies laid the foundation on how to use a large sgRNA library combined with selection pressure to quantify enrichment or depletion of sgRNA, they were constrained by the inability to capture cell specific responses and multi regulatory effects. With a new wave of single-cell sequencing came a transformative shift in functional genomics. A combination of barcoding, single-cell sequencing and pooled CRISPR libraries could now be used for a richer interpretation of gene perturbations beyond binary outcomes.

#### Entry of single-cell RNA sequencing

Perturb-seq was the earliest reported assay that combined massively parallel single-cell RNA sequencing with CRISPR-based multi locus gene perturbations. CRISP-seq, demonstrated to probe regulatory circuits of innate immunity, was also established around the same time as a broadly applicable approach to integrate single-cell RNA sequencing with pooled CRISPR screening.^78^ In a foundational study, Dixit et al. applied Perturb-seq to both primary post-mitotic immune cells and proliferating cell lines.^79^ The authors constructed a custom barcoded sgRNA library targeting 24 immune transcriptional factors to infect bone-marrow-derived dendritic cells. Using a customized-droplet-based single-cell RNA sequencing platform, they sequenced each cell’s transcriptome along with its associated sgRNA. This allowed for direct linkage of each perturbation to its transcriptional phenotype. This was distinct from all other CRISPR screens performed so far because for the first time CRISPR perturbation could be analyzed quantitatively and mechanistically at a single level.

Pooled CRISPR screens that used bulk RNA sequencing had one major limitation. They were unable to distinguish distinct perturbations that were either caused by similar responses from the population of cells or driven by a subset of cells. To interpret the data, the team developed a computational framework, named multi-input multi-output single-cell analysis (MIMOSCA). This program could decipher the effect of individual perturbations and the marginal contributions of genetic interactions at the level of each transcript, program, and cell state.^79^ This framework was compatible with CRISPRi and CRISPRa screens as well as with barcoded cells used for lineage tracing, as demonstrated by a companion study.^80^

#### Simplifying gRNA capture

These developments were followed by CROP-seq, the key advantage being that gRNA could be read directly.^81^ The technology had now moved from indirect gRNA capture to direct gRNA capture. Lenti-Guide-Pro (plasmid popular for pooled CRISPR screens) was re-engineered, which allowed the sgRNA to be co-transcribed and captured during standard single-cell RNA sequencing workflows. CROP-seq thereby solved the challenge of detecting gRNAs in single-cell transcriptomes at the vector level, which made it compatible with various single-cell RNA sequencing assays.^81^ These studies demonstrated that CRISPR perturbations could be paired with single-cell transcriptomic data at a scalable rate.

#### Integrating epigenetic and epitope profiling with Perturb-seq

Subsequent advancements further extended the Perturb-seq framework to multi-omic readouts. For example, Perturb-ATAC combined effects of CRISPR perturbations with single-cell ATAC-seq to profile changes in the epigenome. Pooled CRISPR perturbations with genome-wide chromatin accessibility profiling revealed that genomic co-localization and co-expression of transcription factors may predict genetic interaction.^82^ Yet another extension was Perturb-CITE seq, which paired CRISPR perturbation with simultaneous single-cell RNA profiling and epitope sequencing.^83^ Mosaic-seq was developed to measure one direct phenotype of enhancer repression in single cells.^84^ The contribution of enhancers to target gene expression was being studied at a large-scale single-cell level for the first time. Importantly, this study demonstrated that a reporter or phenotypic selection is not required for the assay.^84^

#### Commercialization of single-cell sequencing technology

Despite the advancement in these techniques, the widespread use of the assay was limited due to the costs and the insurmountable amount of data associated to the single-cell sequencing. Additionally, genes expressed at low levels could not be robustly detected. In that regard, the emergence of TAP-seq (targeted Perturb-seq) enabled the amplification of gene of interest rather than the whole transcriptome. This increased the sensitivity and lowered the cost. Moreover, TAP-seq was compatible with standard 3′ single-cell library preparation methods and implemented for 10× Genomics and Drop-Seq.^85^ This method bypassed the need for engineered polyadenylated barcodes increasing efficiency in high-throughput screens.

Direct-capture Perturb-seq emerged as a key milestone.^86^ It enabled today’s well-known concept of “multiplexing” by targeting individual genes with multiple sgRNA and captured their expression alongside single-cell transcriptomes. This approach eliminated the need for engineered constructs by incorporating capture sequences into the sgRNA scaffold, allowing 5′ and 3′ direct capture on commercial platforms like 10× Genomics. The cost of Perturb-seq experiments was significantly reduced after the introduction of hybridization-based target enrichment demonstrated in this study.^86^ A major turning point came with the commercialization of feature barcoding by 10× Genomics. This innovation provided gel beads embedded with primers designed to capture sgRNAs (or barcoded antibodies) alongside transcriptomic data. This standardized the workflow, reduced costs via target enrichment, and dramatically expanded the accessibility of single-cell pooled CRISPR screening.

A newer advancement, OPS (optical pooled screening), shifted the readout from sequencing to high-resolution imaging.^87^ OPS combines CRISPR perturbation with live-cell imaging and *in situ* barcode detection. This allows spatial, morphological, and dynamic phenotypes such as subcellular localization or cell movement to be measured in parallel with perturbation identity. The group demonstrated a loss-of-function CRISPR screen targeting 952 genes to identify regulators of p65 translocation utilizing the power of pooled live cell imaging.^87^ Today, several other vendors like Parse Biosciences offer alternatives to feature barcoding workflows of 10× Genomics. The integration of CRISPR perturbations with single-cell sequencing technologies further exemplifies CRISPR as a sequencing-coupled platform, where nucleic acid barcoding strategies enable the linkage of specific genetic perturbations to transcriptional phenotypes at single-cell resolution.

#### Next-generation single-cell sequencing modalities

**Figure 1:**
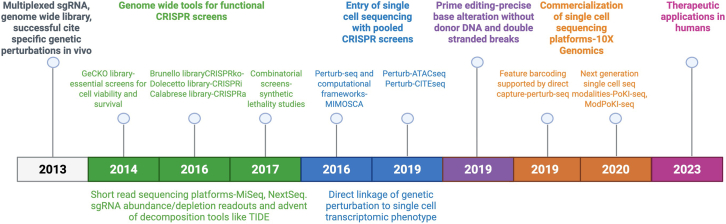
Major milestones in the development of CRISPR-based therapeutics, highlighting key advancements from early genome editing strategies to emerging precision and personalized interventions

A third wave of single-cell CRISPR functional genomics is now emerging in engineered immune cells, especially T cells expressing therapeutic receptors such as TCRs (T cell receptors) and CARs (chimeric antigen receptors). T cell exhaustion has been implicated in the failure of cancer immunotherapy, and improving T cell function is a high-priority area.^88^ Thus, engineering T cells for a therapeutic response is a promising strategy to improve therapy outcome. Genetically reprograming T cells with viral vectors had resulted in unspecific targeting. To overcome limitations of viral delivery and random integration, PoKI-seq (pooled knockin sequencing) was developed. This approach combined single-cell transcriptome analysis and pooled knockin screening to measure cell abundance and cell state *ex vivo* and *in vivo*, accelerating discovery of non-viral knockin programs for cell therapies.^89^ An evolution of this approach, ModPoKI (modular pooled knockin), enables testing of thousands of synthetic DNA sequences (enhancers, promoters, and coding variants) at specific genomic loci alongside TCRs and CARs.^90^ These advances highlight the dramatic progression of CRISPR screening technologies, from basic dropout assays to multi-omic, spatially resolved, and programmable single-cell platforms. As techniques such as ModPoKI, OPS, and PoKI-seq mature, we keep gaining unprecedented control over genome editing and phenotyping, accelerating discoveries in functional genomics, disease modeling, and therapeutic engineering (Figure 1).

### The emergence of CRISPR as an inter-disciplinary field of science

The advent of pooled CRISPR screens combined with single-cell sequencing has generated unprecedented volumes of data. However, the sheer complexity and scale of these data make it challenging to extract meaningful biological insights. Tasks such as assigning each cell to its corresponding gRNA perturbation, analyzing differential effects across thousands of perturbations, and detecting lowly expressed genes, all present significant analytical hurdles. While classical differential expression methods can be applied to both bulk and single-cell CRISPR screen data, more robust and specialized frameworks have emerged to accommodate the unique demands of these high-throughput datasets.

The earliest and foundational framework to estimate the impact of gene perturbations was MIMOSCA.^79^ The primary software on 10× Genomics namely Cell Ranger and Cell Ranger Arc provided steps for demultiplexing sgRNA sequences, aligning reads, and assigning perturbations to individual cells. Complementary to these, the Loupe browser is a primary starting point for researchers for visualizing outputs such as cluster inspection, sgRNA distribution, and differential expression exploration. While these tools do not offer statistical modeling capabilities, they serve as essential entry points for data exploration, particularly when used in conjunction with 10×’s feature barcoding technology for CRISPR-based single-cell assays.

These outputs can be exported for deeper analysis into frameworks developed by Dr. Rahul Satija’s lab (e.g., Mixscape)^91^ that seamlessly integrate with 10×’s data. Python-based ecosystems such as Scanpy have also facilitated advanced analysis pipelines for Perturb-seq workflows, supporting sgRNA assignment, dimensionality reduction, clustering, and visualization (e.g., UMAP).^92^ Over time, several computational frameworks such as scMAGeCK,^93^ SCEPTRE,^94^ MUSIC,^95^ BAGEL,^96^ CRISPhieRmix,^97^ and many more were developed. These tools have been adapted for a variety of CRISPR modalities, knockout, activation, and interference and rely either on direct count matrices or on external preprocessing pipelines to obtain guide-level quantifications before performing gene-level inference. A comprehensive review on the computational frameworks applied to single-cell sequencing of pooled CRISPR screens has been compiled in recent reviews.^98^^,^^99^ More recently, PerturbSci-Kinetics provides end-to-end processing, quality filtering, assignment of perturbation barcodes, and differential analysis.^100^

The accessibility of these tools whether as GitHub repositories, R/Bioconductor packages, or Python modules has been instrumental in democratizing the analysis of CRISPR single-cell datasets. Without these analytical innovations, the biological data produced by such screens would remain largely uninterpretable.

Ultimately, the power of CRISPR as a technology lies not just in its biological applications but in its seamless integration with data science, mathematics, and statistics. The rise of pooled single-cell CRISPR screens has fostered a truly interdisciplinary field, where computational and experimental scientists collaborate to decode cellular function at unprecedented resolution. This interplay, in our opinion, is a defining feature of the CRISPR revolution and a compelling fourth reason why the technology continues to resonate across domains as diverse as biology, chemistry, computer science, and applied mathematics.

### CRISPR and the changing face of science: From lab Bench to public trust

#### Landmark clinical trials in humans

The significance of the technology had reached unprecedent heights, enough for the technology to take its first step in humans. Despite the advancement in the technology, off-target editing by CRISPR-Cas9 remained the main challenge for clinical application. In a landmark study demonstrating the safety and feasibility of genetic editing in humans, a clinical trial in China approved the editing of *PD-1* T cells in advanced non-small cell lung cancer patients in 2016, and the study was reported in 2020.^101^ *PD-1*-edited T cells were manufactured by T cell isolation from PBMCs (peripheral blood mononuclear cells) of patients followed by *ex vivo* expansion and CRISPR-Cas9-mediated knockout of exon 2 of the *PD-1* gene. Researchers hypothesize that disruption of *PD-1* gene could be therapeutic, given that anti-*PD-1* checkpoint inhibitors had become the standard of care in advanced non-small cell lung cancer. Unfortunately, the study did not result in any substantial clinical benefit that could be attributed to the low editing efficiency and lack of T cell expansion. However, the study was designed to demonstrate safety and feasibility of the technology for human intervention. The mutation frequency of off-target effects was fairly low, with the majority of the mutations unlikely to have a significant effect on coding genes. Most importantly, the persistence of the edited T cells up to 4 weeks in all patients supported the efficacy of gene-edited T cells. Furthermore, after a median follow-up of 47.1 weeks, none of the patients developed any severe adverse events related to the treatment.

As a proof of principle, another study reported the successful allogeneic transplantation and long-term engraftment of CRISPR-Cas9-edited, *CCR5*-ablated HSPCs (hematopoietic stem and progenitor cells) in a patient with HIV-1 infection and acute lymphoblastic leukemia.^102^ *CCR5* null blood cells are largely resistant to HIV entry. Although the researchers achieved only 5% disruption of lymphocytes by the introduction of the edited *CCR5* cells, the cells carrying the ablated *CCR5* persisted for more than 19 months without any adverse effects related to gene editing.

Similarly, a phase I clinical trial introduced genetically edited T cells and a *NY-ESO-1* to improve anti-tumor immunity in two patients with refractory myeloma and one with metastatic sarcoma. They reported no clinical toxicities and the persistence of engineered T cells for at least 9 months.^103^ Taken together, these pilot trials successfully demonstrated that multiplex human genome engineering can be safe.

#### Genetic editing in wrong hands

Importantly, these pilot trials aimed at editing somatic cells that did not impact germline cells. The rapid advancement of CRISPR-based genome editing has raised profound ethical considerations, particularly as the technology transitions from experimental systems to clinical applications. A central ethical distinction lies between somatic and germline genome editing. While somatic editing targets non-reproductive cells and affects only the treated individual, germline editing introduces heritable changes that can be transmitted to future generations, raising fundamental concerns regarding consent and long-term safety.^104^ This could be problematic if the consequences were to involve any adverse effects or off-target effects, which was expected with CRISPR-Cas9 engineering.

In an unexpected turn of events, announcement of the first genetically edited babies surfaced at the Second International Summit on Human Genome Engineering Hong Kong in 2018. The researcher expected to confer HIV resistance by editing *CCR5* gene in an IVF embryo and later transplanted the embryos that resulted in the birth of twins. This was the first demonstration of genetic editing highlighting the dangers of premature clinical application in the absence of robust ethical oversight and regulatory approval. Given the unresolved safety concerns that genetic editing raises, international committees proposed regulatory frameworks for performing safe clinical gene editing.^105^

In addition to safety, issues of equity and access are increasingly recognized as central ethical challenges. The high cost and technical complexity of CRISPR-based therapies raise concerns about unequal access and the potential exacerbation of existing healthcare disparities. Furthermore, the possibility of non-therapeutic genome editing, including genetic enhancement, has prompted debate regarding the ethical boundaries of human intervention and the risk of societal stratification based on genetic traits.^104^ In response to these concerns, several international organizations have established guidelines and governance frameworks for the responsible use of genome editing technologies. Reports from World Health Organization established a governance framework on human genome editing along with recommendations on mechanisms for human genome editing.^106^ Similarly, the International Call for a 10-Year Moratorium on Heritable Human Genome Editing outlines safe and responsible use of genetic technologies, emphasizing on a principal-based approach to heritable human genome editing.^107^ Collectively, these ethical and regulatory considerations underscore the need for a cautious and responsible approach to CRISPR-based genome editing. As the technology continues to evolve, ongoing dialogue between scientists, clinicians, policymakers, and the public will be essential to ensure that its clinical implementation aligns with both scientific and societal values.

Despite the lack of regulatory standards during the birth of the twins, public reaction, however, was not nearly as severe as the public outcry that came into effect after the first IVF technology. The initial cohort of the first IVF trials only had 23 women^108^ after the birth of Louis Brown—the first IVF baby in 1978.^109^ IVF too was once greeted with fear, moral concerns, and skepticism. Yet, by the early 2000s, it had become a mainstream, widely acceptable medical procedure, illustrating how societal perception can evolve alongside scientific progress. Despite the controversies surrounding the birth of CRISPR twins, people had a higher acceptance of scientific solutions that researchers provide today.

#### Applications of CRISPR in curing human diseases

**Figure 2:**
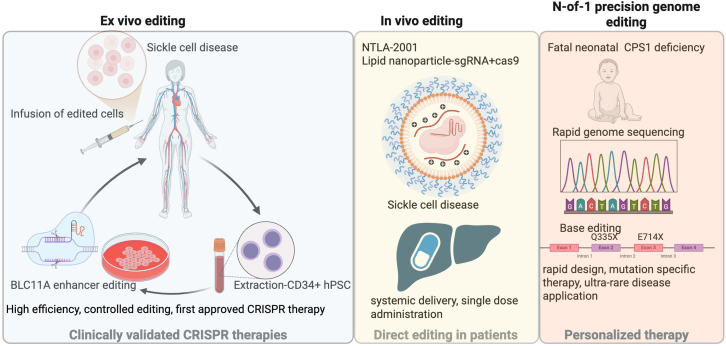
Evolution of CRISPR-based therapies toward precision and individualized genome editing. Created in BioRender. Banik, I. (2025)

The application of CRISPR technology in human disease has rapidly evolved from proof-of-concept studies to clinically validated therapies (Figure 2). This progression can be broadly categorized into established clinical successes, ongoing human trials, and emerging next-generation interventions that are redefining the scope of precision medicine.

#### Clinical successes

A prime example of CRISPR’s utility in clinical success is the development of DETECTR—a diagnostic test for the detection of SARS-CoV-2.^34^ In 2020, Mammoth Biosciences, co-founded by CRISPR pioneer Dr. Jennifer Doudna, released a white paper outlining a detailed protocol for the rapid detection of SARS-CoV-2 within 30 min from sample to result. They designed Cas12 preloaded guides targeting two viral genes (N and E) and implemented RT-LAMP isothermal amplification to facilitate rapid detection without thermal cycling. The assay was highly specific, fast, and did not require specialized lab infrastructure, leading to a swift approval by the Food and Drug Administration (FDA) for detection of patient cases.

In a landmark study demonstrating a durable remission of sickle cell disease using CRISPR-Cas9-mediated gene therapy marketed as Casgevy, faith in the good of science was further restored. The enhancer region of *BCL11A* was targeted in CD34+ HPSCs and infused in patients with transfusion-dependent β-thalassemia (TDT) and sickle cell disease (SCD).^110^ This one-of-a-kind gene therapy is a one-time treatment leading to long-term remission.

#### Human trials

In parallel, a growing number of clinical trials are exploring CRISPR-based interventions across a range of diseases, including cancer, infectious diseases, and genetic disorders, the most notable being in liver-targeted disease. Transthyretin amyloidosis, also called ATTR amyloidosis, is characterized by accumulation of misfolded transthyretin (TTR) protein and is a progressive fatal disease. Patients are expected to have a median lifespan of 2–6 years after the diagnosis.^111^ Prior therapies like patisiran included a reduction of amyloid formation through stabilization of TTR or inhibition of TTR protein synthesis.^112^ These treatments were limited by the long-term administration to maintain the knockdown of TTR. As an alternative to mRNA-targeting-based gene silencing, CRISPR-Cas9 can be used for genome editing. This disease can now be treated by NTLA-2001, which comprises a lipid nanoparticle encapsulating a messenger RNA for Cas9 protein and an sgRNA targeting *TTR*.^113^ It is a single-dose intravenous infusion intended to edit TTR in hepatocytes, leading to a reduction in wild-type and mutant TTR protein. The method of delivery used in NTLA-2001 is a proprietary lipid nanoparticle delivery system. Given that TTR is produced exclusively in the liver, the nanoparticle-mediated delivery of the CRISPR module is expected to maximize efficacy and minimize toxicity. Other examples of the current clinical landscape using CRISPR-Cas9 for therapeutic applications of human disease have been reported in a different study.^114^

#### Next-generation interventions

More recently, the development of personalized CRISPR-based therapies has introduced a new paradigm in precision medicine. A notable example is the treatment of a 6-month-old infant with carbamoyl phosphate synthetase 1 (*CPS1*) deficiency, a rare urea cycle disorder associated with high neonatal mortality. Rapid genome sequencing identified two truncating variants in *CPS1*: c.1003C→T (p.Gln335Ter; Q335X) on the paternal allele and c.2140G→T (p.Glu714Ter; E714X) on the maternal allele. To functionally assess these variants, ∼100 bp genomic regions spanning each mutation were cloned into lentiviral reporter constructs and introduced into HuH-7 cells. Following transcription, the impact of each variant on RNA processing was evaluated using sequencing-based analysis of transcript isoforms, enabling quantitative comparison of wild-type and mutant sequences and revealing variant-specific effects on gene expression. Based on these findings, a personalized therapeutic strategy was developed using base editing to correct the pathogenic mutation at the DNA level. A customized lipid nanoparticle delivery system, analogous to that used in NTLA-2001, was employed to deliver the editing machinery *in vivo*. The patient received two infusions—a low dose at 7 months and a higher dose at 8 months of age—and showed marked clinical improvement within weeks, including increased protein tolerance and improved metabolic stability during illness.^115^ No other clinical cases with *CPS1* deficiency treated with CRISPR-Cas9-mediated gene therapy have been reported. This case represents the first demonstration of a personalized *in vivo* CRISPR-based therapy designed to correct an individual’s genetic mutation and one of the earliest applications of base editing in a pediatric patient. More broadly, this “N-of-1” approach highlights the feasibility of rapidly integrating sequencing, functional validation, and targeted nucleic acid editing to develop therapies for ultra-rare genetic disorders, a paradigm that was previously unattainable using conventional drug development frameworks.

Collectively, these advances illustrate a clear trajectory in the clinical application of CRISPR—from *ex vivo* editing of accessible cell types to systemic *in vivo* interventions and, ultimately, to individualized therapies. Therapeutic applications of CRISPR depend on the delivery and activity of nucleic acid components, including gRNAs and editing templates, reinforcing the central role of nucleic acid engineering in both experimental and clinical contexts. As delivery technologies, editing precision, and regulatory frameworks continue to improve, CRISPR is poised to expand its impact across a broader spectrum of human diseases, including those that have historically been considered intractable. In less than a decade, CRISPR has progressed from a genome engineering tool to a clinically validated therapeutic platform, marking one of the fastest translational trajectories in modern biomedical science.

CRISPR beyond human interventions: the extraordinary cures developed with CRISPR have not been limited to human applications. For instance, wheat was genetically edited to reduce free asparagine by knocking out the *TaASN2* (asparagine synthetase 2) gene using CRISPR-Cas9.^116^ This was one of many reports that demonstrated that targeted editing could achieve trait improvements without yielding fitness penalties, distinct from random mutagenesis and transgenics. Furthermore, the approval for such recent crop trials that incorporated CRISPR technology exemplifies how European regulations are shifting to accommodate gene-edited crops. As an experimental proof of concept in arthropod genetics, the first reports of CRISPR-modified spiders spinning red fluorescent dragline silk have been published.^117^ Although experimental, this is a leap in the field of material science since spider silk is a sought-after product for strong natural fibers. As another notable example, Colossal Biosciences works exclusively on the representation of de-extinction and re-integration of critically endangered animal projects through CRISPR and related genome editing techniques. Although not entirely perfect examples of true de-extinction, they have achieved multiple live births of Woolly Mice^118^ and Red Wolves^119^ using CRISPR for either insertion of genes or genetic correction.

#### Limitations and challenges

One of the most widely recognized limitations is off-target editing, where RNA-guided endonuclease (RGN) induce mutations other than intended genomic loci. Potential off-target activity could occur with three to five base pair mismatches in the PAM-distal part of the sgRNA guiding sequence.^120^ Delivery of Cas9 protein and sgRNA directly into cells compared to delivery of plasmids encoding Cas9 and sgRNA has resulted in reduced off-target effects.^121^^,^^122^ Some CRISPR systems also use NRG sequence instead of NGG as PAM sequence, which are known to have low cleavage efficiency.^123^ Off-targeting could have serious consequences in the host organism such as chromosomal rearrangements and loss of function of gene activity causing physiological abnormalities.^124^ Significant progress has been made to mitigate these challenges. Advances in tools to design efficient sgRNA and minimize off-targets such as Cas-Off Finder, CRISPR Design, E-CRISP, and CRISPRdirect have been reported.^125^ Other strategies include increasing the specificity of target site, decreasing the expression of nuclease expression for minimizing accumulation of off-target mutations, direct delivery of Cas9 complex, inducible Cas9 systems, and truncated sgRNAs.^124^ In parallel, the emergence of genome-wide detection methods such as GUIDE-seq, Digenome-seq, SITE-seq, GOTI, and CIRCLE-seq has enabled more accurate identification and avoidance of off-target sites.^125^ Collectively, these innovations suggest that while off-target effects remain a critical concern, they are increasingly predictable and, to a large extent, controllable in well-optimized systems.

CRISPR technologies can be viewed as a unified class of nucleic-acid-guided platforms that integrate programmable targeting with sequencing-based and biochemical readouts. This convergence has enabled unprecedented scalability in functional genomics, accelerated translational applications, and continues to redefine the interface between molecular biology, computation, and medicine. Overall, CRISPR has opened avenues for thriving innovators contributing to aspects of science that are unconventional and distinct from mainstream academia or biotech-industry-oriented goals. Therefore, it would suffice to say that the technology has raised a scientific culture that paces with the faith of the common people and the understanding that it is indeed for the betterment of the society. In our view, this growing public trust is the fifth and the most powerful reason CRISPR is entering mainstream discourse—not only in science but also in popular imagination. Although the technology is still battling challenges, emerging platforms that use artificial intelligence like Evo2^126^ may be used to predict the outcomes of genome editing or better predict off-target effects. Ultimately, the CRISPR revolution signals a new era—one where the line between intervention and innovation blurs and where the phrase “playing God” is being redefined as a pursuit of healing, resilience, and understanding.

## Acknowledgments

We sincerely thank Dr. David Adams for his guidance, insights, and feedback on the write-up of this review article. This review article did not receive any funding.

## Author contributions

I.B., concept, research, and writing; J.-P.C., reviewing and editing.

## Declaration of interests

The authors declare no competing interests.
